# Clinical Outcomes of Patients with AmpC-Beta-Lactamase-Producing Enterobacterales Bacteremia Treated with Carbapenems versus Non-Carbapenem Regimens: A Single-Center Study

**DOI:** 10.3390/antibiotics13080709

**Published:** 2024-07-29

**Authors:** Orjowan Shalabi, Livnat Kashat, Omer Murik, Shoshana Zevin, Marc V. Assous, Eli Ben-Chetrit

**Affiliations:** 1 Medical Department B, Shaare Zedek Medical Center, The Eisenberg R&D Authority, Hadassah Medical School, Hebrew University, Jerusalem 91904, Israel; 2 Faculty of Medicine, Hebrew University, Jerusalem 91904, Israel; 3 Clinical Microbiology Lab, Shaare Zedek Medical Center, Hadassah Medical School, Hebrew University, Jerusalem 91904, Israel; 4 Translational Genomics Lab, Medical Genetics Institute, Shaare Zedek Medical Center, Jerusalem 91904, Israel; 5 Infectious Diseases Unit, Shaare Zedek Medical Center, The Eisenberg R&D Authority, Hadassah Medical School, Hebrew University, Jerusalem 91904, Israel

**Keywords:** AmpC, bacteremia, carbapenem, ciprofloxacin

## Abstract

Introduction: Bloodstream infections caused by AmpC-producing Enterobacterales pose treatment challenges due to the risk of AmpC overproduction and treatment failure. Current guidelines recommend carbapenems or cefepime as optimal therapy. We aimed to evaluate empiric and definitive non-carbapenem regimens for these infections. Methods: In a retrospective study from June 2014 to March 2023, adult bacteremic patients with *Enterobacter cloacae* complex strains and *Morganella morganii* were evaluated. Demographic, clinical and lab data and outcomes were assessed. Results: The cohort comprised 120 bacteremic patients, 17 receiving empiric carbapenem and 103 non-carbapenem regimens. Both groups had similar Charlson and Norton scores and previous antimicrobial exposure. The most common sources of bacteremia were urinary, abdominal and central-line-associated sources. Empiric non-carbapenem regimens (*primarily* piperacillin–tazobactam and cephalosporins) were not associated with recurrent bacteremia or 30-day mortality. Definitive regimens included mainly carbapenems (*n* = 41) and ciprofloxacin (*n* = 46). Beta-lactams were administered to 25 patients. Recurrent bacteremia and 30-day mortality rates were similar among treatment groups. Ciprofloxacin showed comparable outcomes to carbapenems, however, severity of illness among these patients was lower. Conclusions: Empiric and definitive non-carbapenem regimens for bacteremia with AmpC-producing organisms were not associated with treatment failure or increased 30-day mortality. Ciprofloxacin appears promising for selected, stable patients, potentially enabling early discharge.

## 1. Introduction

Bloodstream infections with wild-type AmpC-beta-lactamase-producing Enterobacterales pose a therapeutic challenge. AmpC-beta-lactamase is a cephalosporinase capable of hydrolyzing all penicillins and cephalosporins except cefepime. AmpC expression is inducible in response to beta-lactam exposure, although non-inducible chromosomal resistance (promoter and/or attenuator mutations) and *ampC* plasmid-mediated resistance have also been reported [1,2,3]. Since treating AmpC-producing pathogens with β-lactams can induce AmpC overproduction and subsequent β-lactam resistance, even in infections initially caused by susceptible isolates, the optimal management of these infections remains controversial. The magnitude of production may be affected by the type of β-lactam and species [4]. First-generation cephalosporins are potent inducers [5]; *Enterobacter* species demonstrate high-AmpC production, in contrast to *Morganella morganii* isolates of which AmpC expression is considered to be low [6].

While some studies consider carbapenems as the mainstay treatment [7], others tend to recommend carbapenem-sparing agents, using beta-lactam/beta-lactamase inhibitor combinations or cefepime [8]. The choice is also affected by the source of infection (high- versus low-inoculum infection) and source control [9]. A recent study discouraged the use of piperacillin/tazobactam due to higher early treatment failure rates as compared with carbapenems [10]. In contrast, a recent meta-analysis by Simone et al. showed no difference in 30-day mortality rates between carbapenem versus non-carbapenem regimens administered to treat AmpC-producing Enterobacterales bloodstream infections [11].

In the Merino II prospective trial, it has been shown that treatment with piperacillin/tazobactam was associated with more microbiological failure (i.e., recurrent bacteremia on days 3–5 post-randomization) compared to treatment with carbapenem for AmpC-producing Enterobacterales (5/38 versus 0/34, respectively, *p* = 0.03). However, 30-day mortality rates were similar between patient groups and the study was limited by its sample size [12].

Nevertheless, current Infectious Diseases Society of America (IDSA) guidance suggests cefepime or carbapenems as the recommended treatment for infections with AmpC-producing Enterobacterales, aside from cases of uncomplicated cystitis of which third-generation cephalosporins may be a reasonable option [13].

In this study, we aimed to examine demographic and clinical characteristics and outcomes of bacteremic patients with AmpC-producing pathogens treated with carbapenems versus non-carbapenem regimens.

## 2. Results

Blood cultures positive for Enterobacter spp. or *Morganella morganii* were identified in 169 patients during the study period: 14 patients were excluded as they died within 48 h of the positive blood culture, 25 patients were excluded due to prolonged admission (>30 days) as well as 10 same-day duplicate blood cultures from patients (duplicate samples). The final cohort of 120 bacteremic patients included 84 with *Enterobacter* species (*Enterobacter cloacae* complex, primarily *Klebsiella aerogenes*) and 36 with *Morganella morganii* (Figure S1).

Patients who had been treated empirically with carbapenems (*n* = 17) versus non-carbapenem regimens (*n* = 103) prior to the appearance of the AmpC bacteria were compared with regard to demographic, clinical and lab characteristics (Table 1). The two groups had similar baseline demographic characteristics. The majority of patients resided at home and were subsequently admitted to medical wards (*n* = 96, 79.2%). Charlson and Norton’s scores were similar for both groups as was previous exposure to antimicrobial therapy during 90 days prior to admission (Table 1).

*Enterobacter cloacae* complex strains were the most prevalent bloodstream isolates in both groups, followed by *Morganella morganii*. Ceftriaxone resistance was demonstrated in 25/120 isolates (*Enterobacter* species, *n* = 21/84 (25%)) and was significantly associated with prior exposure to antibiotics (OR 5.3 (95% CI 1.8–15.2), *p* = 0.001).

The most common likely source of bacteremia in the cohort was the urinary tract followed by abdominal infection and central-line-associated bacteremia (Table 2). Source control was achieved in both groups. Severity of illness (Pitt bacteremia score) and need for vasopressor/inotropic support were comparable between the groups.

**Figure 1:**
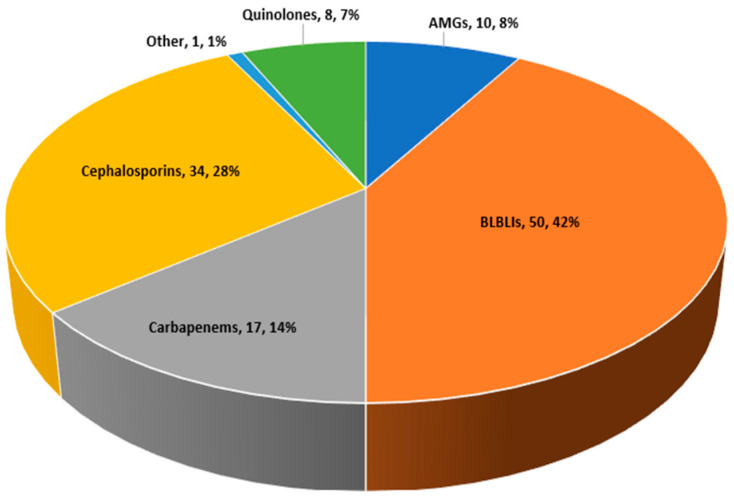
Distribution of *empiric* antimicrobial regimens among 120 cohort patients. AMGs, aminoglycosides. BLBLIs, beta-lactam/beta-lactamase inhibitors.

The non-carbapenem regimen included mostly a beta-lactam/beta-lactamase inhibitor (BLBLI) (*n* = 50, 42%), followed by cephalosporins (*n* = 34, 28%) (Figure 1).

Overall, inappropriate empiric antimicrobial therapy (based on in vitro susceptibility) was administered to 19 patients (16%) (Table 2). Once the isolate was identified and antimicrobial susceptibility testing (AST) was available, the antibiotic regimen was modified in 72/73 (99%) patients in the empiric non-carbapenem group (versus 1/17 (5.9%) in the carbapenem group) (Table 3). In these cases, the median time to definitive treatment was 2 days (IQR 2–3). Empiric antimicrobial treatment with a non-carbapenem regimen was not associated with persistent or recurrent bacteremia, admission to ICU, or increased 30-day mortality (Table 3).

**Figure 2:**
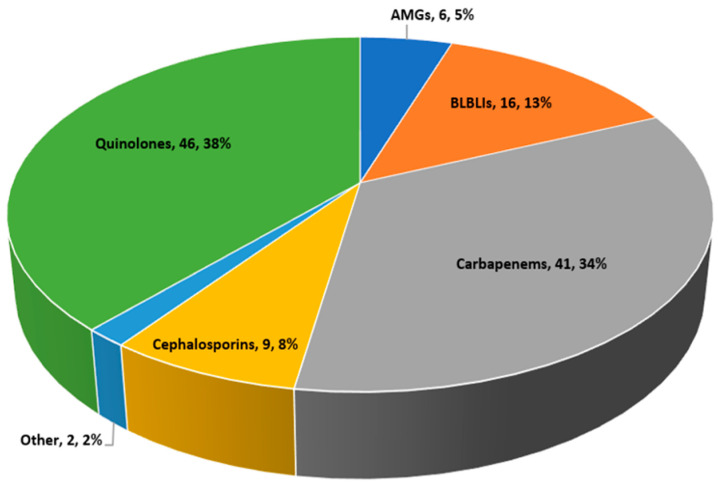
Distribution of *definitive* antimicrobial regimens among 120 cohort patients. AMGs, aminoglycosides. BLBLIs, beta-lactam/beta-lactamase inhibitors.

Figure 2 shows the distribution of *definitive* antimicrobial regimens. A shift towards treatment with either a carbapenem or ciprofloxacin was noted. Definitive treatment with a BLBLI or third-generation cephalosporins was administered to 25 patients (piperacillin–tazobactam, *n* = 16, third-generation cephalosporins, *n* = 9). Of these, only one bloodstream isolate was resistant to ceftriaxone.

Baseline demographic and clinical characteristics were similar between the two groups (Table 4 and Table S1). Resistance to ceftriaxone was significantly associated with a switch to definitive carbapenem treatment (OR 3.2, 95% CI 1.3–7.9, *p* = 0.01). Additionally, definitive carbapenem treatment was more likely to be administered to patients with a higher Pitt bacteremia score (2.7 ± 2.9 versus 1.6 ± 2.1, *p* = 0.02) and a higher CRP level (20.6 ± 11.2 versus 16.6 ± 8.7, *p* = 0.04) (Table 4).

On multivariate analysis, Pitt bacteremia score and ceftriaxone resistance were both found to be significantly associated with definitive treatment with a carbapenem (OR 1.2, 95% CI 1.01–1.4 and 2.9, 95% CI 1.2–7.4, respectively, *p* < 0.05). Since a ceftriaxone resistance phenotype, when observed, was a clear indication to administer a carbapenem, a subgroup analysis was conducted on bacteremic patients with ceftriaxone-susceptible isolates. The Pitt bacteremia score continued to be associated with definitive carbapenem treatment (OR 1.3, 95% CI 1.06–1.64, *p* = 0.01).

Ciprofloxacin was frequently used as definitive therapy (*n* = 46, 38%) (Figure 2). Patients who were treated with ciprofloxacin versus non-ciprofloxacin regimens had lower CRP levels and lower Pitt bacteremia scores (1.5 ± 2.3 versus 2.2 ± 2.5, *p* = 0.04, Table S2).

Table 5 shows the clinical outcomes of patients with regard to definitive therapy. All outcome measures, including persistent bacteremia, recurrent bacteremia, and 30-day mortality, were similar among patients treated with either a *definitive* carbapenem or a non-carbapenem regimen.

The median durations of treatment (empiric and definitive) among patients in the carbapenem and non-carbapenem regimens groups were comparable (9 days, IQR 8–10, versus 9, IQR 7–11, respectively, *p* = 0.8).

On univariate analysis investigating factors linked to mortality, elevated Pitt bacteremia score and requirement of vasopressors/inotropes found they were significantly correlated with increased 30-day mortality (*p* < 0.05) (Table 6). A trend towards higher mortality was noted with regard to non-urinary sources of infection (*p* = 0.09). Ceftriaxone resistance, *Enterobacter* species versus *Morganella morganii* infection, non-carbapenem empiric or definitive regimens, as well as inappropriate empiric therapy were not associated with increased 30-day mortality. In an adjusted analysis, elevated Pitt bacteremia score remained significantly associated with mortality (*p* = 0.0006) (Table 6).

Regarding length of hospitalization, after excluding patients who died during admission (*n* = 15), the Pitt bacteremia score was the only variable associated with increased length of stay (*p* = 0.03).

Examining the subgroup of patients who were treated with ciprofloxacin versus carbapenems as definitive therapy showed similar outcomes including 30-day mortality (5/46 (11%) in the ciprofloxacin group versus 7/41 (17%) in the carbapenem group, *p* = 0.53) (Figure S2). Ciprofloxacin was administered orally in 32/46 patients (70%). Among patients treated with oral ciprofloxacin versus carbapenems, a trend towards a shorter admission was noted (13.3 ± 8.2 versus 15.7 ± 6.7, respectively, *p* = 0.19).

## 3. Discussion

In this study, we evaluated the clinical characteristics and outcomes of bacteremic patients with *Enterobacter cloacae* complex strains and *Morganella morganii*, who were treated empirically with carbapenems versus non-carbapenem regimens prior to identification of the resistant organism. Non-carbapenem empiric regimens predominantly comprised BLBLIs followed by cephalosporins (Figure 1). Adjusted analysis showed that antimicrobial treatment with non-carbapenem regimens (empiric or definitive) was not significantly associated with persistent bacteremia, recurrent bacteremia, ICU admission and increased 30-day mortality.

The optimal treatment of AmpC-producing Enterobacterales is controversial. IDSA guidance recommends either a carbapenem or cefepime (if MIC < 4 ug/mL) for high-mutation-rate isolates such as *Enterobacter cloacae*, *Klebsiella aerogenes* and *Citrobacter freundii* [6,13]. Third-generation cephalosporins (for susceptible isolates) are suggested in cases of low-mutation-rate pathogens such as *Morganella morganii* or *Serratia marcescens* and for low-inoculum infections (e.g., urinary tract infections). No clear recommendation exists for treatment with quinolones, except when oral step-down is considered and susceptibility is demonstrated.

In the present cohort, neither empiric nor definitive treatment with a non-carbapenem agent was associated with increased treatment failure or 30-day mortality compared with carbapenems. Despite a high proportion of high-mutation-rate isolates (*Enterobacter cloacae* complex strains (*n* = 84/120)), where 71/84 patients (85%) were treated *empirically* with a *non*-carbapenem agent (Table 2), only 4/103 (3.9%) events of treatment failure occurred. However, these findings should be interpreted with caution due to the small sample size of the *empiric* carbapenem group (*n* = 17 versus 103). Although demographic and clinical characteristics were quite similar between the groups (Table 1 and Table 2), the imbalance in group sizes may have affected our results. Nevertheless, these findings are consistent with previous reports. In a cohort of 458 AmpC-producing Enterobacterales bloodstream infections, empiric therapy with piperacillin–tazobactam or third-generation cephalosporins was not associated with increased mortality risk compared to cefepime, carbapenem and non-beta-lactam therapy [1]. Other studies examining cefepime or piperacillin–tazobactam versus carbapenems showed no difference in terms of mortality or length of stay [2,9]. In a study by Lim et al. the 30-day mortality rate among 110 patients with ESBL and AmpC bacteremia episodes was 20% with no correlation with inappropriate empirical antibiotics, and there was no significant mortality difference between carbapenem use in empirical and definitive therapy [14]. The above-noted results contrast with older studies where infection with high-mutation-rate pathogens was associated with more breakthrough bacteremia events and increased 30-day mortality [15,16]. Moreover, a recent report examining 575 patients with AmpC-producing pathogens (mainly pneumonia and bacteremia) found that AmpC-related treatment failure remained more common in patients receiving third-generation cephalosporins (15% vs. 1%) or piperacillin (6% vs. 1%) compared with reference therapy (carbapenems or cefepime) even in patients infected with low-mutation-rate pathogens such as *Morganella morganii*. One major limitation of that study was that, in cases of pneumonia, treatment failure relied on the managing physician’s differentiation between colonization and infection [17].

The overall low rate of treatment failure in the present study may be attributed to several important factors: a relatively high rate of urosepsis (27%) representing low-inoculum infections, adequate source control, infrequent ceftriaxone-resistant isolates (*n* = 25/120, 21%) and the frequent use of carbapenems or quinolones as definitive therapy consistent with IDSA guidance [13]. Once AST results were available, there was a notable shift towards treatment with either carbapenems or ciprofloxacin as definitive therapy. Indeed, ceftriaxone resistance was associated with definitive treatment with carbapenems. However, the decision on the definitive antimicrobial regimen was likely influenced by the severity of illness (i.e., higher Pitt bacteremia score) regardless of AST results (Table 4).

The frequent use of ciprofloxacin as a definitive treatment warrants consideration. Recent retrospective studies support switching to oral fluoroquinolones after different intravenous antibiotic treatments, with no increase in clinical failure rates, although longer treatment duration (not necessarily in hospital) was noted [18,19,20]. Gunter et al. [21] showed that patients with AmpC-producing Enterobacterales bacteremia who were treated with fluoroquinolones had lower treatment failure rates compared to patients treated with beta-lactam antibiotics (12.9% vs. 24%). These patients also had lower Pitt bacteremia scores, similar to the patients in our study who were eventually treated with ciprofloxacin (Table S2). In the present cohort, clinical outcomes, including treatment failure and 30-day mortality, were comparable among patients treated with ciprofloxacin versus carbapenems as definitive regimens, and a non-significant trend towards shorter length of stay was noted among patients who were switched to oral ciprofloxacin. However, these findings should be interpreted with caution due to limited sample size and variations in severity of illness.

Finally, when evaluating factors associated with 30-day mortality, univariate and adjusted analysis showed that only Pitt bacteremia score correlated with increased 30-day mortality (Table 6). This finding is not surprising and aligns with previous reports [22,23,24,25]. Non-urinary source infections may have also contributed to increased mortality, reflecting severe or high-inoculum infections as has been previously proposed [13,23,24,25], although in the present study, only borderline significance was noted (Table 6).

Our study has several limitations. First, the retrospective design was prone to selection bias, especially regarding decisions on empiric and definitive antimicrobial therapies. Second, as noted above, the sample size of the empiric carbapenem group was rather small, limiting the interpretation of the findings regarding empiric treatment. Once definitive treatments were prescribed, the groups were more balanced. Third, only *Enterobacter* species and *Morganella morganii* bloodstream isolates were included, excluding other AmpC-producing agents. However, these agents represent high- and low-mutation-rate pathogens well. Lastly, definitive non-carbapenem regimens primarily included ciprofloxacin, limiting the assessment of piperacillin–tazobactam or third-generation cephalosporins as definitive treatments due to sample size. However, this allowed for the evaluation of ciprofloxacin as a definitive treatment in bacteremia with AmpC-producing agents, for which the literature is scarce.

In summary, empiric and definitive treatments with non-carbapenem regimens among bacteremic patients with AmpC-producing agents were not significantly associated with recurrent bacteremia or increased 30-day mortality. Among selected, stable patients, ciprofloxacin appears to be a suitable option and may allow early discharge.

## 4. Materials and Methods

This retrospective single-center observational study examined cases of bloodstream infection with *Enterobacter cloacae complex* and *Morganella morganii,* from June 2014 to March 2023, that were retrieved from the computerized microbiological database. These strains were selected as they represent high- and low-mutation-rate AmpC-producing organisms. Cases were limited to adult patients aged 18 years or older.

Currently, there are no Clinical and Laboratory Standards Institute (CLSI)-endorsed criteria for AmpC detection in clinical isolates. Most tests that identify AmpC production demonstrate a phenotype of possible resistance to beta-lactamase antibiotics by using gradient strips consisting of cloxacillin and cefotetan/cefoxitin, boronic acid disks added to cephamycin disks (double-disk synergy test) or disk potentiation tests [7,10]. In the SZMC microbiology lab, the possibility of AmpC resistance is first considered among typical isolates (i.e., *Enterobacter cloacae*). ESBL resistance, which may coexist with AmpC resistance, is ruled out using double disk diffusion synergy tests (ceftazidime/clavulonate and cefotaxime/clavulonate synergy test) [26]. If the resistance mechanism remains unclear, a synergy test with and without cloxacillin is performed (cloxacillin is an inhibitor of AmpC-producing species but not ESBL). For cases where resistance to carbapenems is observed, a carbapenmase test is performed and the resistance mechanism is usually detected by lateral flow assays and/or real-time PCR.

In this study, all *Enterobacter cloacae* and *Morganella morganii* isolates harboring additional resistance mechanisms other than AmpC were excluded. Other exclusion criteria included prolonged admission (longer than 30 days) and death within 48 h of admission.

Demographic, clinical and laboratory data were collected for each patient with bacteremia, including age, gender, Charlson score, Pitt bacteremia score (day of bacteremia), Norton score, prior exposure to antibiotics during the previous 6 months, presumed source of bacteremia based on discharge diagnosis and clinical course, source control, empirical antibiotic treatment and definitive antibiotic regimens. Laboratory data, including complete blood count, liver and renal function tests and C-reactive protein (CRP) were also recorded.

Outcome measures such as length of hospitalization, ICU admission, recurrent or persistent bacteremia and 30-day mortality were documented. Persistent bacteremia was defined as repeat positive blood cultures with the same pathogen ≥ 72 h after the administration of any antimicrobial agent. A repeat positive blood culture was considered identical to the initial culture if [1] the same organism was isolated and [2] the antibiogram (in vitro susceptibility profile) matched that of the initial culture. Recurrent bacteremia was defined as a repeat positive blood culture with the same pathogen ≥ 48 h after withdrawal of antibiotics and up to 30 days after the day of bacteremia. Any case of persistent or recurrent bacteremia was considered treatment failure.

Descriptive statistics summarized the demographic, clinical and lab characteristics, as well as outcome measures of bacteremic patients treated with carbapenems versus non-carbapenem regimens. Correlation between treatment outcomes and quantitative variables (including clinical and lab parameters) was assessed using the *t*-test, for two independent treatment groups, and the Mann–Whitney test for non-normal distribution of the tested variable. For categorical variables, a *χ*^2^ test or Fisher’s exact test was used. Variables found to be statistically significant (two-sided *p*-value < 0.05) in a univariate analysis for the dependent variable (e.g., definitive carbapenem treatment, 30-day mortality) were further examined using both a stepwise forward likelihood ratio and multivariate logistic regression model.

All statistical analyses were carried out using Ep info™ 7 (CDC) and SPSS software (version 20).

## 5. Conclusions

Empiric and definitive non-carbapenem regimens for bacteremia caused by AmpC-producing organisms were not associated with treatment failure or increased 30-day mortality. Ciprofloxacin appears promising for selected, stable patients, potentially enabling early discharge.

## Figures and Tables

**Table 1:** Baseline demographic and clinical characteristics of study patients treated empirically with carbapenems and non-carbapenem antimicrobial regimens.

Parameter	All Patients (*n* = 120)	*Empiric* Carbapenems, *n* = 17	*Empiric* Non-Carbapenem Regimens, *n* = 103	*p* Value
Age (years), mean ± SD	72.3 ± 14.9	67.5 ± 15.1	73.1 ± 14.8	0.15
Female, n (%)	50 (41.7)	9 (53)	41 (40)	0.4
Basic metabolic index (BMI) ^a^, mean	26.6 ± 5.2	25.2 ± 4	26.9 ± 5.3	0.2
Residence, n (%) Home/assisted living care home Nursing home	103 (85.8) 17 (14.1)	14 (82.4) 3 (17.7)	89 (86.4) 14 (13.6)	0.7
Department Medical Surgical Critical care	95 (79.2) 19 (15.8) 6 (5)	14 (82.3) 2 (11.8) 1 (5.9)	81 (78.6) 17 (16.5) 5 (4.9)	0.8
Charlson score, mean ± SD	4.2 ± 2.7	3.4 ± 2.9	4.3 ± 2.6	0.7
Norton score, mean ± SD	14.4 ± 4.8	15.8 ± 4.8	14.2 ± 4.8	0.2
Previous exposure to antimicrobial therapy (90 days)	61 (50.8)	10 (58.8)	51 (49.5)	0.6

^a^ Missing data, *n* = 2, carbapenem group; *n* = 6, non-carbapenem group.

**Table 2:** Clinical and laboratory characteristics of study patients empirically treated with carbapenems versus non-carbapenem antimicrobial regimens.

Variable, *n* (%)	All Patients (*n* = 120)	*Empiric* Carbapenems, *n* = 17	*Empiric* Non-Carbapenem Regimens, *n* = 103	*p* Value
Pathogen *Enterobacter cloacae* complex *Morganella morganii*	84 (70) 36 (30)	13 (76.5) 4 (23.5)	71 (68.9) 32 (31.1)	0.6
Likely source of bacteremia Urosepsis Abdominal Endovascular/CRBSI Bone and soft tissue Pneumonia Febrile neutropenia Other/undetermined ^a^	32 (26.7) 27 (22.5) 26 (21.7) 14 (11.7) 9 (7.5) 7 (5.8) 5 (4.2)	4 (23.5) 7 (41.2) 4 (23.5) 0 0 2 (11.8) 0	28 (27.2) 20 (19.4) 22 (21.4) 14 (13.6) 9 (8.7) 5 (4.9) 5 (4.9)	1 0.06 1 0.2 0.4 0.3 1
Source control	57/83 (67.7)	11/15 (73.3)	46/68 (67.7)	0.8
Systolic blood pressure < 90 mmHg	44 (36.7)	5 (29.4)	32 (37.9)	0.6
Inotropic support	25/120 (20.8)	4/17 (23.5)	21/103 (20.4)	0.8
Pitt bacteremia score, mean ± SD	1.9 ± 2.4	1.9 ± 2.5	2 ± 2.4	0.9
White blood cell count < 4000/uL or >12,000/uL	66 (55)	10 (58.8)	56 (54.4)	0.8
C-reactive protein (mg/dL), mean ± SD ^b^	17.9 ± 9.7	17 ± 11.5	18 ± 9.5	0.7
Creatinine level ^c^	2.3 ± 2.3	1.8 ± 1.4	2.4 ± 2.4	0.6
Creatinine above 1.5 ^d^	52/115 (45.2)	8/17 (47)	44/98 (44.9)	1
Lactate, mean ± SD ^e^	3.1 ± 2	2.7 ± 1.5	3.2 ± 2	0.5
Inappropriate empiric therapy ^f^	19 (15.8)	2 (11.7)	17 (16.5)	1

^a^ One patient had post-surgical meningitis and was treated with meropenem. The source of bacteremia was undetermined in five patients who were treated empirically with a non-carbapenem regimens. ^b^ Non-carbapenem regimen, *n* = 92/103, carbapenems, *n* = 13/17. ^c^ Day of bacteremia. ^d^ Excluding five hemodialysis patients. ^e^ Non-carbapenem regimens, *n* = 58/103, carbapenems, *n* = 10/17. ^f^ Based on in vitro susceptibility testing.

**Table 3:** Clinical outcomes of patients treated *empirically* with carbapenems versus non-carbapenem regimens.

Variable, *n* (%)	*Empiric* Carbapenems (*n* = 17)	*Empiric* Non-Carbapenem Regimens (*n* = 103)	*p* Value
Modification of empiric treatment *	1 (5.9)	72 (70)	<0.001
Length of stay **	15.5 ± 7.2	14.5 ± 7.6	0.6
Admission to ICU within 14 days	2 (11.8)	16 (15.5)	1
Persistent bacteremia	0	2 (2)	NA
Recurrent bacteremia	0	4 (3.9)	NA
30-day mortality	1 (5.9)	14 (13.6)	0.6

* Modification of antimicrobial regimens included switching the empiric regimen to carbapenems (*n* = 33), ciprofloxacin (*n* = 34), aminoglycosides (*n* = 2) and other antibiotics (*n* = 4). ** Excluding patients who died during admission (*n* = 15).

**Table 4:** Clinical and laboratory characteristics of study patients treated with *definitive* carbapenems versus non-carbapenem antimicrobial regimens.

Variable, *n* (%)	*Definitive* Carbapenems, *n* = 41	*Definitive* Non-Carbapenem Regimens, *n* = 79	*p* Value
Pathogen *Enterobacter cloacae* complex *Morganella morganii*	30 (0.73) 11 (26.8)	54 (68.3) 25 (31.7)	0.7
Likely source of bacteremia Urosepsis Abdominal Endovascular/CRBSI Bone and soft tissue Pneumonia Febrile neutropenia Undetermined ^a^	12 (29.3) 9 (22) 11 (26.8) 5 (12.2) 2 (4.9) 1 (2.4) 1 (2.4)	20 (25.3) 18 (22.8) 15 (19) 9 (11.4) 7 (8.9) 6 (7.6) 4 (5)	0.7 0.9 0.3 0.9 0.6 0.3 0.7
Source control	23/30 (76.7)	34/53 (64.2)	0.3
Systolic blood pressure < 90 mmHg	17 (41.5)	27 (34.2)	0.4
Inotropic support	12 (29.3)	13 (16.5)	0.15
Pitt bacteremia score, mean ± SD	2.7 ± 2.9	1.6 ± 2.1	0.02
White blood cell count < 4000/uL or >12,000/uL	22 (53.7)	44 (55.7)	0.8
C-reactive protein (mg/dL), mean ± SD ^b^	20.6 ± 11.2 (*n* = 35)	16.6 ± 8.7 (*n* = 70)	0.04
Creatinine level ^c^	2.7 ± 2.3	2.1 ± 2.2	0.2
Creatinine above 1.5 ^d^	20/37 (38.5)	32/78 (61.5)	0.2
Lactate, mean ± SD ^e^	3.3 ± 2.5 (*n* = 25)	3 ± 1.6 (*n* = 43)	0.5
Ceftriaxone resistance	14/41 (34%)	11/68 (14%)	0.016

^a^ One patient had post-surgical meningitis and was treated with meropenem. The source of bacteremia was undetermined in four patients who were treated with a non-carbapenem regimens. ^b^ CRP level was available in 35 and 70 patients in the definitive carbapenem and definitive non-carbapenem treatment groups, respectively. ^c^ Day of bacteremia. ^d^ Excluding hemodialysis patients (*n* = 5). ^e^ Lactate level was available in 25 and 43 patients in the definitive carbapenem and non-carbapenem treatment groups, respectively.

**Table 5:** Clinical outcomes of patients treated with *definitive* carbapenems versus non-carbapenem regimens.

Variable, *n* (%)	*Definitive* Carbapenems (*n* = 41)	*Definitive* Non-Carbapenem Regimens (*n* = 79)	*p* Value
Length of stay *	15.7 ± 6.7	14.1 ± 7.9	0.3
Admission to ICU within 14 days	9 (22)	9 (11.4)	0.18
Persistent bacteremia	1 (2.4)	1 (1.3)	1
Recurrent bacteremia	1 (2.4)	3 (3.8)	1
30-day mortality	7 (19.5)	8 (12.7)	0.38

* Excluding patients who died during admission (*n* = 15).

**Table 6:** Risk factors associated with 30-day mortality, univariate and multivariate, adjusted analysis.

	Univariate Analysis			Multivariate Analysis		
Term	OR	95% CI	*p* Value	OR	95% CI	*p* Value
Age	1.02	0.98–1.06	0.36			
Female gender (Yes/No)	1.3	0.4–3.7	0.68			
Previous 90 d admission (Yes/No)	1.9	0.57–6.4	0.3			
Previous antimicrobial therapy	1.1	0.38–3.3	0.8			
Charlson score	1.2	0.97–1.4	0.1			
Norton score	0.97	0.86–1.09	0.6			
Cognitive impairment (Yes/No)	0.3	0.04–2.4	0.3			
Pitt bacteremia score	1.4	1.2–1.7	0.0005	1.4	1.2–1.8	0.0006
Inotropic support (Yes/No)	5.9	1.9–18.4	0.002			
WBC > 12,000 or <5000	2.5	0.75–8.4	0.1			
CRP	1.05	0.99–1.1	0.12			
Kidney injury *	0.8	0.26–2.4	0.7			
Lactate above 4 (*n* = 68)	1.5	0.3–6.6	0.6			
Ceftriaxone resistance	0.94	0.25–3.6	0.9			
Empiric carbapenems vs. non-carbapenem regimens	0.4	0.05–3.2	0.4			
Definitive carbapenems vs. non-carbapenem regimens	1.8	0.6–5.5	0.28			
Definitive treatment with carbapenems vs. ciprofloxacin **	0.6	0.17–2	0.53			
Inappropriate empiric antimicrobial therapy (based on in vitro AST)	1.4	0.4–6.5	0.4			
Mean time to definite therapy	1.3	0.9–1.8	0.13			
*Enterobacter* sp. (versus *Morganella morganii*)	0.4	0.15–1.3	0.14			
Ceftriaxone resistance	0.94	0.25–3.6	0.93			
Non-urinary source infection	5.9	0.74–46.6	0.09	7.3	0.8–64.7	0.08
Source control	0.9	0.2–3.9	0.9			

* Cre above 1.5 mg/d, day of bacteremia. ** Definitive carbapenem treatment, *n* = 41; definitive ciprofloxacin treatment, *n* = 46. AST, antimicrobial susceptibility testing.

## Data Availability

The data presented in this study are available on request from the corresponding author due to ethical restrictions.

## Supplementary Materials

The following supporting information can be downloaded at: https://www.mdpi.com/article/10.3390/antibiotics13080709/s1, Figure S1: Study cohort flow chart; Figure S2: Kaplan–Meier survival analysis of definitive carbapenem- versus ciprofloxacin-treated patients; Table S1: Baseline demographic characteristics of study patients treated with *definitive* carbapenems and non-carbapenem antimicrobial regimens; Table S2: Clinical and laboratory characteristics of study patients treated with *definitive* carbapenems versus ciprofloxacin. Table S3: Individual patient data table.
