# Supplementary material for: Clinical Outcomes of Patients with AmpC-Beta-Lactamase-Producing Enterobacterales Bacteremia Treated with Carbapenems versus Non-Carbapenem Regimens: A Single-Center Study
Source: Antibiotics (Basel). 2024 Jul 29;13(8):709. doi: 10.3390/antibiotics13080709 (PMC11350690; doi:10.3390/antibiotics13080709)
Supplement: Supplementary file 1 [file antibiotics-13-00709-s001.zip › antibiotics-3093905 - SM.pdf]

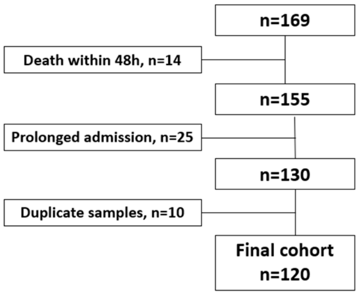

Figure S1. Study cohort flow chart.

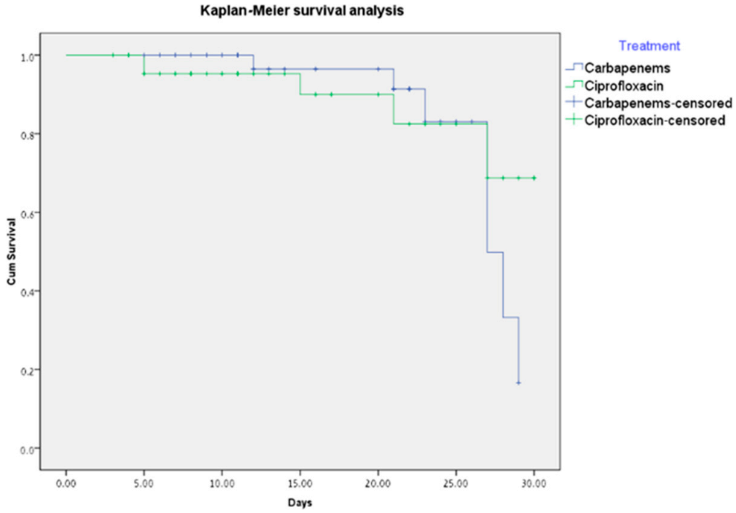

Figure S2. Kaplan-Meier survival analysis of definitive carbapenem- versus ciprofloxacin-treated patients.

Table S1. Baseline demographic characteristics of study patients treated with *definitive* carbapenems and non-carbapenem antimicrobial regimens.

**Parameter**	***Definitive* carbapenems, n=41**	***Definitive* non-carbapenem regimens, n=79**	***p*-Value**
Age (years), mean±SD	71.1±16.4	73±14	0.5
Female, n (%)	18 (43.9)	32 (40.5)	0.84
**Residence, n (%)**			
Home / assisted living care-home	32 (78)	71 (89.9)	0.1
Nursing home	9 (22)	8 (10.1)	
**Department**			
Medical	32 (78)	63 (79.7)	0.5
Surgical	3 (7.3)	13 (16.5)	
Critical care	6 (14.6)	3 (3.8)	
Charlson score, mean±SD	4±2.8	4.3±2.6	0.7
Norton score, mean±SD	13.6±4.8	14.8±4.8	0.2
Previous exposure to antimicrobial therapy (90 days)	23 (56)	38 (62)	0.4

IQR, interquartile range; SD, standard deviation.
