# Supplementary material for: Clinical Outcomes of Patients with AmpC-Beta-Lactamase-Producing Enterobacterales Bacteremia Treated with Carbapenems versus Non-Carbapenem Regimens: A Single-Center Study
Source: Antibiotics (Basel). 2024 Jul 29;13(8):709. doi: 10.3390/antibiotics13080709 (PMC11350690; doi:10.3390/antibiotics13080709)
Supplement: Supplementary file 1 [file antibiotics-13-00709-s001.zip › Table S2.pdf]

**Table S2.** Clinical and laboratory characteristics of study patients treated with *definitive* carbapenems versus ciprofloxacin.

| **Variable, n (%)** | ***Definitive* carbapenems, n=41** | ***Definitive* ciprofloxacin, n=46** | ***p* value** |
|---|---|---|---|
| Age, mean±SD | 71.1±16.4 | 71.1±14.2 | 1 |
| Pathogen | | | |
| *Enterobacter species* | 30 (73.2) | 36 (78.3) | 0.6 |
| *Morganella morganii* | 11 (26.8) | 10 (21.8) | |
| Non-urinary source of bacteremia | 29 (70.7) | 33 (71.7) | 1 |
| Source control | 23/30 (76.7) | 16/30 (53.3) | 0.1 |
| Pitt bacteremia score, mean±SD | 2.7±2.9 | 1.5±2.3 | **0.035** |
| White blood cell count < 4,000/uL or > 12,000/uL | 22 (53.7) | 44 (55.7) | 0.8 |
| C-reactive protein (mg/dL), mean±SD[a] | 20.6±11.2 | 15±7.6 | **0.016** |
| Creatinine level (day of bacteremia)[b] | 2.7±2.3 | 2.1±2.1 | 0.2 |
| Ceftriaxone resistance | 14 (34%) | 8 (17.4%) | 0.09 |

[a]C-reactive protein (CRP) level was available in 35 and 42 patients in the definitive carbapenem and ciprofloxacin groups, respectively.

[b]Excluding hemodialysis patients (n=5).
